# Novel sperm selection device on the basis of microfluidics improves usable blastocyst rates, embryo morphology, and morphokinetic patterns: sibling cohort prospective study

**DOI:** 10.1016/j.xfre.2025.09.008

**Published:** 2025-10-06

**Authors:** Fernando Meseguer, Laura Carrión-Sisternas, Ana del Arco, Rocío Rivera-Egea, Carmen Vidal, José Alejandro Remohí, Marcos Meseguer

**Affiliations:** a IVIRMA Global Research Alliance, IVIRMA Valencia, Valencia, Spain; b IVIRMA Global Research Alliance, IVI Foundation, Instituto de Investigación Sanitaria La Fe IIS La Fe, Valencia, Spain; c IVIRMA Global Research Alliance, IVIRMA Madrid, Madrid, Spain

**Keywords:** Microfluidics, sperm selection, Swim-up, DNA fragmentation, outcomes

## Abstract

**Objective:**

To assess the performance of a novel microfluidic sperm selection device, SwimCount Harvester (SCH), compared with the Swim-up (SU) method, analyzing both sperm quality and the laboratory’s key performance indicators.

**Design:**

Prospective, double-blind study using sibling oocytes.

**Subjects:**

This study included 100 patients undergoing intracytoplasmic sperm injection. Both partners had normal karyotypes. Male participants provided only fresh ejaculated semen samples without total asthenozoospermia, globozoospermia, azoospermia, or cryptozoospermia. Female partners had at least 2 mature oocytes retrieved.

**Exposure:**

Semen samples were analyzed to assess concentration, total progressive motile count, vitality, morphology, chromatin structure integrity, and deoxyribonucleic acid fragmentation index (DFI) before and after sperm selection. Subsequently, each sample was divided into two aliquots and processed using either the SCH device or the Swim-up technique. Retrieved oocytes from each patient were divided into two groups and microinjected with sperm from the corresponding preparation method. The injected oocytes were cultured in time-lapse incubators to collect morphokinetic data and apply artificial intelligence (AI)–based tools for assessing oocytes and embryos.

**Main Outcome Measures:**

Primary outcomes included fertilization rate, usable blastocyst rate, good-quality blastocyst rate, euploidy rate, embryo morphokinetics, and AI score to predict pregnancy for each embryo. Secondary outcomes encompassed oocyte quality (using AI tool) and sperm quality parameters.

**Results:**

SwimCount Harvester yielded significantly higher sperm concentration, morphologically normal sperm, improved chromatin stability, and lower DFI. The usable blastocyst rates were higher in the SCH group (SCH, 40.5%, vs. SU, 34.5%), as well as the good-quality blastocyst rates (SCH, 30.8%, vs. SU, 23.4%). Time to blastocyst formation was shorter in the SCH group (SCH, 106.9 hours, vs. SU, 109.5 hours). Fertilization rates (SCH, 78.8%, vs. SU, 77.0%), euploidy rates (SCH, 40.4%, vs. SU, 37.2%), and AI scores to predict pregnancy (SCH, 5.7%, vs. SU, 4.6%) were comparable. Subgroup analyses revealed higher usable blastocyst and good-quality embryo rates in the SCH group among cases with a DFI of >20%, as well as a higher good-quality embryo rate in low-quality oocytes (AI score of ≤6) in the SCH group.

**Conclusion:**

SwimCount Harvester improved sperm quality compared with Swim-up. Furthermore, the SCH device increased the usable blastocyst rate and embryo quality rate and reduced time to blastocyst formation. Nevertheless, no significant differences were observed in the fertilization rates, euploidy rates, or AI scores.

In nature, sperm selection occurs in the female reproductive tract and is a highly selective process (1). The journey of sperm cells from the cervix to the egg is fraught with challenges that affect their viability and motility. These include the acidic environment of the vagina, the dense mucus, convoluted channels of the cervix, and an inflammatory sort of reaction in the uterus. Collectively, these obstacles help ensure that only the healthiest sperm reaches the fallopian tubes (2). However, patients undergoing treatments at assisted reproduction clinics, such as intracytoplasmic sperm injection (ICSI), bypass the natural barriers of the female reproductive tract. Hence, the sperm sample must undergo laboratory processing to isolate the best sperm subpopulation effectively; this procedure is known as sperm selection (3, 4).

Since the beginning of assisted reproductive techniques, sperm selection has been performed using density gradients and Swim-up. Both techniques select spermatozoa by density and motility, respectively (5). However, neither technique can effectively isolate sperm subpopulation with intact deoxyribonucleic acid (DNA) integrity (6). This limitation arises from the fact that both techniques involve subjecting the sample to centrifugation, a process that, according to several studies, can lead to an increase in reactive oxygen species, thereby posing a potential risk of genetic material damage (6, 7). Additionally, certain components of the gradient culture media contain transition metals that have a high affinity for nucleic acids, contributing to DNA fragmentation (7).

In this context, microfluidics has emerged as a promising technology that overcomes many of the limitations of conventional sperm selection methods by mimicking the physiological selection mechanisms present in the female reproductive tract (8). This technology enables the manipulation of small volumes of seminal fluid through microscale channels, allowing for precise control of the physical and chemical microenvironment (9, 10), enabling a less invasive selection process with reduced oxidative stress (8, 11).

Compared with conventional methods, microfluidic devices offer numerous advantages. The elimination of centrifugation reduces both mechanical stress and oxidative stress and allows for the direct processing of unwashed semen samples, minimizing handling time, reagent consumption, and contamination risk. Furthermore, these systems offer scalability, automation, and continuous, controlled culture conditions (8, 11). Several studies have reported significant improvements in sperm motility and morphology, as well as reduced DNA fragmentation, after the use of microfluidic platforms compared with Swim-up and density gradient techniques (9, 12, 13, 14, 15, 16, 17, 18, 19, 20). These findings support the hypothesis that microfluidics can help isolate a sperm subpopulation with higher functional competence. It has also been proposed that this improvement in sperm quality may translate into better laboratory outcomes. Indeed, several studies have investigated the impact of microfluidic sperm selection on fertilization rates, embryo development, and clinical outcomes, reporting favorable results when compared with conventional techniques (17, 21, 22, 23, 24). However, other studies have not observed significant differences in these parameters, highlighting the need for well-designed clinical trials to validate the real-world impact of this technology (25, 26, 27, 28).

Therefore, given the variability reported in existing literature, the present study aimed, for the first time, to conduct the clinical validation of a novel microfluidics-based device for sperm selection. The objectives were to compare sperm quality obtained with the microfluidic device vs. the Swim-up technique and to assess the impact of the sperm selection method on the in vitro fertilization laboratory’s key performance indicators (KPIs).

## Materials and methods

### Study design and population

This prospective, double-blind study using sibling oocytes was conducted at IVI-Valencia. The aim was to compare the performance of the membrane-based microfluidic device SwimCount Harvester (SCH; MotilityCount ApS, Copenhagen, Denmark) with the Swim-up technique for sperm selection in ICSI cycles. The institutional ethics committee approved the study, and all participants provided written informed consent. Assuming a mean of eight mature oocytes per patient and accounting for an estimated 10% dropout rate, the projected sample size for the study was 100 couples. Only fresh ejaculated semen samples without total asthenozoospermia, globozoospermia, azoospermia, or cryptozoospermia were considered eligible. Female participants were over 18 years of age and yielded at least two mature oocytes at the time of oocyte retrieval. All patients had a previously confirmed normal karyotype.

**Figure 1:**
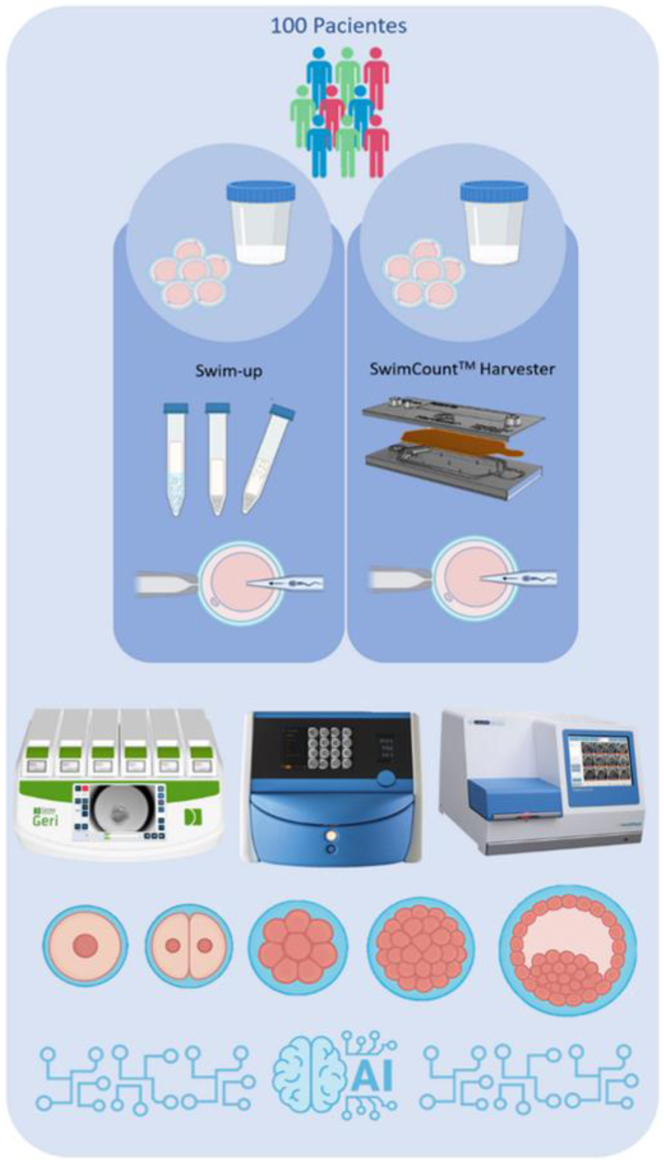
Schematic diagram of the experimental design of the study. A total of 100 patients were included, and each semen sample was divided into 2 equivalent fractions to be processed using 2 sperm selection methods: Swim-up and a membrane-based microfluidic device. After oocyte retrieval, mature oocytes were equally divided into 2 groups. Spermatozoa obtained from each selection method were then used for intracytoplasmic sperm injection into the corresponding groups of mature oocytes. The resulting embryos were cultured in time-lapse incubators and subsequently assessed both morphologically and through morphokinetic parameters of embryo development. In addition, artificial intelligence tools were employed to evaluate oocyte quality and to predict the likelihood of pregnancy of each generated blastocyst.

Each semen sample was divided into two equal aliquots: one processed using the conventional Swim-up method and the other using the SCH device. After oocyte retrieval, half of the mature oocytes were injected with spermatozoa selected via Swim-up, and the other half were injected with spermatozoa processed using the membrane-based microfluidic device, in a double-blind manner. Samples processed by each method were randomly labeled as A or B, ensuring that the embryologists performing the microinjections were blinded to the sperm selection method used for each group. The study design is summarized in Figure 1.

### Semen analysis and sperm selection

Semen samples were collected by masturbation, and a basic semen analysis was performed on fresh samples following the World Health Organization 2021 guidelines. Sperm concentration and motility were assessed using a Makler chamber. Two independent technicians performed the evaluation, each analyzing two separate drops of the sample. Morphology was evaluated with the Bio-Diff stain (Biognost, Zagreb, Croatia) and vitality using the Sperm VitalStain kit (NidaCon International AB, Mölndal, Sweden). Chromatin integrity was analyzed via aniline blue staining (Sigma-Aldrich, St. Louis, MO), and sperm DNA fragmentation index was determined using the terminal deoxynucleotidyl transferase dUTP nick end labeling assay. In this method, DNA breaks were labeled with fluorescein isothiocyanate-labeled dUTP and subsequently analyzed using flow cytometry. The APO-DIRECT kit (BD Pharmingen; BD Biosciences, Franklin Lakes, NJ) was used for this purpose. Samples were fixed in 1% paraformaldehyde for 60 minutes, washed twice with phosphate-buffered saline (Gibco; Thermo Fisher Scientific, Bishops Stortford, England, UK), and stored in 70% ethanol at −20 °C. To perform the analysis, the ethanol was removed from the samples using a washing solution, followed by incubation with a DNA labeling solution (fluorescein isothiocyanate-labeled dUTP) at 37 °C for 60 minutes. Subsequently, the samples were rinsed with the washing solution. Samples were incubated with propidium iodide and RNase for 30 minutes. Negative controls were also prepared, excluding the TdT transferase from the DNA labeling solution to prevent binding of the labeled dUTP to DNA breaks. Finally, using the flow cytometer (CytoFLEX-S; Beckman Coulter Life Science, Indianapolis, IN), the DNA fragmentation of 20,000 spermatozoa was analyzed.

For sperm selection, each semen sample was divided into 2 1-mL aliquots. One aliquot was processed using the Swim-up technique, in which the sample was mixed with an equal volume of culture medium (FertiCult Flushing medium; FertiPro, Beernem, Belgium) and centrifuged at 800 × *g* for 10 minutes. The supernatant was discarded, and the pellet was resuspended in 0.6 mL of Sequential Fert medium (Origio; Cooper Surgical, Ballerup, Denmark). After incubation at 37 °C for 15–30 minutes, the top 0.5 mL, enriched in motile sperm, was collected.

The second aliquot was processed using the SCH device, which was certified with the CE mark. The SCH was composed of two chambers separated by a 10-μm–pore size membrane. One milliliter of semen was loaded into the lower chamber, and 0.8 mL of Sequential Fert medium was loaded into the upper chamber. The device was incubated in a horizontal position at 37 °C for 15–30 minutes, after which the medium from the upper chamber, enriched in highly motile spermatozoa, was carefully collected for further analysis. The device design is shown in Supplemental Figure 1 (available online).

### Ovarian stimulation, embryo oocyte retrieval, and fertilization

Ovarian stimulation was performed according to the routine practice of the clinic (29, 30). Ovulation was triggered using human chorionic gonadotropin (Ovitrelle; Merck & Co., Darmstadt, Germany), gonadotropin-releasing hormone agonist (Decapeptyl; Ipsen Pharma, Boulogne-Billancourt, France), or dual triggering when at least three follicles of ≥18-mm diameter were observed. Oocyte retrieval was performed 36 hours after triggering. Subsequently, oocytes were cultured for 4 hours at 37 °C, 6% CO2, and 5% O2 using a fertilization medium (Origio Sequential Fert, Cooper Surgical). Afterward, oocytes were denudated by pipetting in a 40 IU/mL of hyaluronidase solution. Intracytoplasmic sperm injection was performed under an Olympus IX7 microscope at a magnification of ×400 using gamete medium (Cook Medical, Bloomington, IN).

### Embryo culture and evaluation

Microinjected oocytes were cultured in time-lapse incubators: EmbryoScope (Vitrolife, Gothenburg, Sweden); EmbryoScope Plus (Vitrolife); and Geri (Genea Biomedx, Sydney, Australia). The embryo culture was under stable conditions of temperature (37 °C), CO_2_ (6%), and O_2_ (5%). Fertilization was confirmed 16–18 hours after ICSI. Embryo development was monitored using analysis software EmbryoViewer workstation (Vitrolife) and Geri Connect & Assess2.0 (Genea Biomedx) on an external computer until day 5 or 6 of development. Upon completion of embryo development, morphokinetic parameters were exported. Images of up to 11 focal planes were automatically captured every 10–20 minutes. The division times to 2 cells (t2), 3 cells (t3), 4 cells (t4), and 5 cells (t5) and the time to blastocyst formation were automatically annotated using the guided annotation tool integrated into EmbryoViewer and Geri Connect and Assess2.0, according to the studies by Bori et al. (31) and Alegre et al. (32).

Usable blastocysts were those transferred or vitrified for future use. Blastocyst quality was graded from A to D according to the ASEBIR guidelines (33), with embryos classified as grades A and B considered of good quality. The corresponding grading system is provided in Supplemental Table 1. Additionally, the Magenta software (Future Fertility, Toronto, Ontario, Canada) was used to assess oocyte quality. This artificial intelligence (AI) tool analyzes oocyte images and generates a quality score ranging from 0 to 10, where 0 represents the lowest and 10 the highest oocyte quality. The images analyzed were the first time-lapse capture taken immediately after ICSI. Furthermore, to predict the pregnancy potential of each resulting blastocyst, the Life Whisperer Viability (LWV) (Adelaide, South Australia, Australia) AI tool was used. Blastocyst images were acquired at 120 hours of incubation or at the time of blastocyst formation, in cases where development occurred after the 120-hour point.

### Statistical analysis

The statistical analysis was performed using the Statistical Package for the Social Sciences (Released 2017, IBM SPSS Statistics for Windows, Version 25.0; IBM Corp., Armonk, NY). Semen quality was analyzed using the Wilcoxon signed-rank test for paired samples. The Mann-Whitney *U* test for nonparametric data was applied to compare groups and evaluate potential improvements in laboratory KPIs. Categorical variables were expressed as frequencies and percentages, and differences between groups were analyzed using the chi-square (χ^2^) test. A *P* value of <.05 was considered statistically significant. Furthermore, subgroups were analyzed on the basis of Future Fertility Magenta scores below or above 6, as well as DNA fragmentation levels below or above 20%. For these analyses, the same statistical approach was applied to evaluate differences in fertilization rate, usable blastocyst rate, embryo quality, euploidy rate, morphokinetics, and AI-based viability scores.

## Results

In this prospective study, a total of 100 patients were recruited, with a mean age of 38.23 ± 4.31 years, mean body mass index of 23.72 ± 4.49 kg/m^2^, and mean serum antimüllerian hormone level of 2.75 ± 3.09 ng/mL. A detailed description of the patient characteristics is provided in Supplemental Table 2.

### Comparison of the sperm quality between the SCH and Swim-up groups

Sperm concentration was significantly higher in the SCH group (6.50 × 10^6^/mL; interquartile range [IQR], 1.40–14.00) than in the Swim-up group (3.00 × 10^6^/mL; IQR, 0.83–7.00; Z = −7.434; *P* < .001; r = 0.743). However, progressive motile sperm percentage was slightly higher in the Swim-up group (98.00%; IQR, 94.25–99.00) than in the SCH group (97.00%; IQR, 92.25–99.00; Z = −2.244; *P* = .025, r = 0.224). In contrast, the total progressive motile sperm count was also higher in the SCH group (4.98 × 10^6^; IQR, 1.13–10.00) than in the Swim-up group (2.35 × 10^6^; IQR, 0.65–4.93; Z = −7.412; *P* < .001; r = 0.741). Sperm vitality showed no statistically significant difference between groups (SCH, 95.00% [IQR, 89.00–98.00]; Swim-up, 96.00% [IQR, 90.00–98.00]; Z = −0.194; *P* = .846; r = 0.028). Nevertheless, the proportion of morphologically normal sperm was higher in the SCH group (7.00%; IQR, 5.00–10.00) than in the Swim-up group (6.00%; IQR, 3.25–8.75; Z = −3.289; *P* < .001; r = 0.400). Similar results were obtained when the stability of the chromatin structure was evaluated. The percentage of spermatozoa with correct stability of the chromatin structure showed significantly higher values in the SCH group (90.00%; IQR, 88.00–93.00) than in the Swim-up group (90%; IQR, 84.00–93.00; Z = −2.627; *P* = .009; r = 0.354). Additionally, DNA fragmentation was significantly lower in the SCH group (2.97%; IQR, 1.86–6.72) than in the Swim-up group (5.61%; IQR, 2.39–8.74; Z = −3.271; *P* = .001; r = 0.430).

### Comparison of the oocyte quality between the SCH and Swim-up groups

The assessment of oocyte quality using the AI tool showed no statistically significant differences between the two groups. This finding remained consistent across the global analysis of oocytes, as well as within the subgroups of autologous and donor oocytes, and in the subgroup stratified by DNA fragmentation index. The results are presented in detail in Supplemental Table 3.

### Analysis of the laboratory’s KPIs between the SCH and Swim-up groups

**Figure 2:**
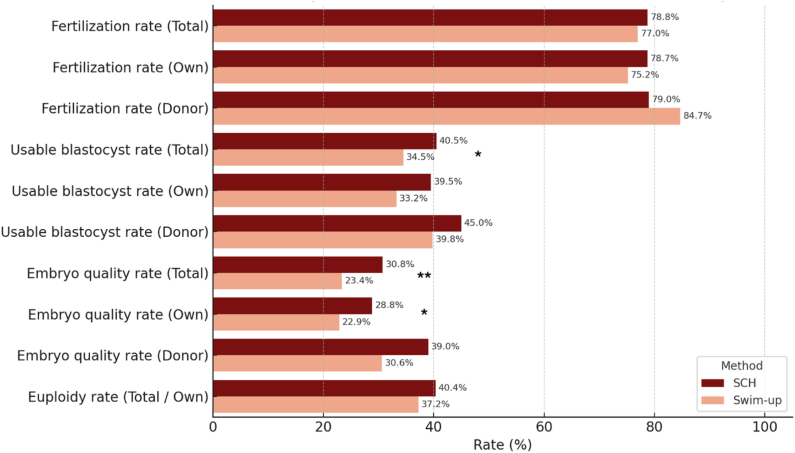
Comparison of fertilization, usable blastocyst, embryo quality, and euploidy rates per mature oocyte or embryo biopsied between SwimCount Harvester (SCH) and Swim-up methods. Bars represent mean outcome rates (%) across all cases, subdivided into total cohort, own oocytes, and donor oocytes where applicable. Dark red indicates SCH-derived outcomes; light red indicates Swim-up. Values are shown atop each bar. This comparative visualization highlights clinically relevant performance trends between conventional and microfluidic sperm selection techniques.

In the analysis of the full sample (n = 100 patients), conducted without subgroup stratification, no statistically significant differences in fertilization rates were found between the SCH group (78.78%) and the Swim-up group (77.00%) (χ^2^ = 0.476, *P* = .490). The usable blastocyst rate per mature oocyte was significantly higher for the SCH group (40.54%, 212/523) than for the Swim-up group (34.50%, 177/513) (χ^2^ = 4.019, ;*P* = .045). Similarly, the microfluidic group demonstrated a significantly higher rate of good-quality blastocysts per mature oocyte, achieving 30.78% (161/523), compared with 23.39% (120/513) in the Swim-up group (χ^2^ = 7.159, *P*=.007). Conversely, the euploidy rate per embryo biopsied was comparable for both groups (SCH, 40.38%, 42/104, vs. Swim-up, 37.23%, 35/94; χ^2^ = 0.206; *P*=.650). In addition, the time to reach a blastocyst was statistically significantly shorter for the SCH group than for the control group (*P*=.036). Finally, no differences were observed in the scores provided by the AI-based embryo evaluation system between the two groups (*P*=.189). Figure 2 and Table 1 provide a detailed overview of the outcomes.

**Table 1:** Comparison of in vitro fertilization laboratory Key Performance Indicators (KPI) between the SwimCount Harvester and Swim-Up groups according to oocyte origin.

Morphokinetic parameters and AI score	Subgroup	n (SCH)	SCH	n (SU)	SU	*P* value
t2 Median (h) (IQR)	Total	388	26.33 (23.87–28.51)	378	26.23 (24.09–28.10)	.9159
	Own	309	26.40 (24.19–28.68)	297	26.35 (24.28–27.96)	.6457
	Donor	79	25.44 (23.27–27.77)	81	25.45 (23.35–28.91)	.5595
t3 Median (h) (IQR)	Total	381	36.40 (32.25–39.50)	368	36.48 (33.10–39.06)	.7883
	Own	305	36.59 (32.60–39.55)	288	36.50 (33.02–38.96)	.4609
	Donor	76	34.67 (31.46–39.46)	80	36.35 (33.45–40.70)	.0384^a^
t4 Median (h) (IQR)	Total	378	37.89 (34.80–41.39)	365	38.07 (35.10–40.84)	.8496
	Own	302	38.21 (34.95–41.55)	286	38.14 (35.24–40.35)	.6879
	Donor	76	36.98 (34.46–40.61)	79	38.00 (34.41–42.00)	.261
t5 Median (h) (IQR)	Total	366	47.96 (43.26–53.26)	358	48.24 (42.79–52.50)	.5432
	Own	293	48.27 (43.50–53.48)	280	48.38 (42.45–52.50)	.2371
	Donor	73	46.67 (41.20–52.00)	78	47.70 (43.56–52.91)	.2971
tB Median (h) (IQR)	Total	260	106.95 (101.25–115.10)	232	109.46 (103.30–115.69)	.036^a^
	Own	207	107.80 (101.79–115.30)	190	109.71 (103.19–116.69)	.175
	Donor	53	103.70 (100.07–112.08)	42	108.43 (104.43–114.05)	.044^a^
Embryo AI score (LWV) Median (IQR)	Total	271	5.70 (1.40–8.00)	240	4.60 (1.27–7.40)	.1892
	Own	217	5.00 (1.40–7.70)	194	4.65 (1.30–7.30)	.6336
	Donor	54	7.20 (3.58–8.73)	46	4.20 (1.28–7.70)	.0549

*Note:* AI = artificial intelligence; IQR = interquartile range; LWV = Life Whisperer Viability; SCH = SwimCount Harvester; SU = Swim-up.

a Morphokinetic parameters (t2–tB) denote the timing (in hours) of key embryonic developmental events recorded by time-lapse imaging. The LWV score indicates the embryo’s predicted probability of achieving pregnancy, as determined by an artificial intelligence–based assessment. Comparisons between the SCH and SU groups were performed using the Mann–Whitney *U* test, stratified by oocyte origin. *P* < .05 was considered statistically significant.

The database was stratified into two groups according to the oocyte quality score generated by the AI tool. In the subgroup with an AI score of ≤6, the use of the SCH device only significantly improved embryo quality per microinjected mature oocyte (*P*=.018). In contrast, within the subgroup with AI-derived scores above 6, no significant differences were observed between the two groups across any of the variables analyzed. The outcomes are thoroughly outlined in Supplemental Table 4.

In a separate analysis, the database was stratified into two groups according to the sperm DNA fragmentation index. When analyzing the group of patients with DNA fragmentation above 20%, no differences in fertilization rates were observed between the two groups. However, the usable blastocyst rate per mature oocyte was higher with SCH (38.82% [33/85]) than with Swim-up (22.67% [17/75], *P*=.028). Likewise, embryo quality per mature oocyte improved significantly when using the microfluidic device, with 34.11% (29/85) of good-quality embryos for the SCH group and 13.33% (10/75) for the Swim-up group (*P*=.002). On the contrary, no significant differences were found for the euploidy rate between the two groups. Similarly, no significant differences were found in embryo morphokinetics and AI score for the pregnancy prediction. Nevertheless, in the group of patients with DNA fragmentation below 20 %, no statistically significant differences were observed in any of the variables analyzed. Table 2 presents a comprehensive summary of the findings.

**Table 2:** Comparison of in vitro fertilization laboratory Key Performance Indicators (KPI) between the SwimCount Harvester and Swim-up groups according to the sperm deoxyribonucleic acid fragmentation index.

Laboratory KPI	Subgroup	SCH	SU	χ^2^	*P* value
Fertilization rate per mature oocyte % (n/N)	<20% DFI	78.07% (292/374)	77.25% (292/378)	0.205	.651
	≥20% DFI	80.00% (68/85)	74.67% (56/75)	0.650	.420
Usable blastocyst rate per mature oocyte % (n/N)	<20% DFI	41.98% (157/374)	36.77% (139/378)	2.135	.144
	≥20% DFI	38.82% (33/85)	22.67% (17/75)	4.841	.028^a^
Embryo quality rate per mature oocyte % (n/N)	<20% DFI	30.48% (114/374)	25.93% (98/378)	1.927	.165
	≥20% DFI	34.11% (29/85)	13.33% (10/75)	9.337	.002^a^
Euploidy rate per embryo biopsied % (n/N)	<20% DFI	13,54% (31/229)	12,23% (28/229)	0.175	.676
	≥20% DFI	20.83% (5/24)	13.64% (3/22)	0.414	.520

Morphokinetic parameters and AI score	Subgroup	n (SCH)	SCH	n (SU)	SU	*P* value
t2 Median (h) (IQR)	<20% DFI	183	25.50 (23.49–27.52)	166	25.50 (23.78–27.11)	.962
	≥20% DFI	44	27.13 (24.51–29.50)	32	27.10 (24.63–28.44)	.771
t3 Median (h) (IQR)	<20% DFI	183	36.40 (32.77–39.00)	166	36.20 (33.80–38.10)	.860
	≥20% DFI	44	36.75 (32.67–41.99)	32	36.25 (32.85–39.32)	.603
t4 Median (h) (IQR)	<20% DFI	183	37.40 (34.59–40.16)	166	37.30 (35.17–39.36)	.941
	≥20% DFI	44	39.11 (35.65–43.02)	32	39.13 (34.77–42.03)	.947
t5 Median (h) (IQR)	<20% DFI	183	48.03 (43.90–52.87)	166	49.11 (44.54–52.15)	.747
	≥20% DFI	44	48.05 (43.82–53.82)	32	50.22 (46.34–54.92)	.546
tB Median (h) (IQR)	<20% DFI	183	106.90 (102.09–115.10)	166	109.15 (103.62–115.65)	.135
	≥20% DFI	44	108.57 (100.30–115.56)	32	113. 20 (105.74–116.88)	.290
Embryo AI score (LWV) Median (IQR)	<20% DFI	183	5.90 (1.90–8.10)	163	5.00 (2.03–7.80)	.194
	≥20% DFI	44	5.90 (1.10–7.95)	32	2.85 (0.98–6.25)	.282

*Note:* AI = artificial intelligence; DFI = deoxyribonucleic acid fragmentation index; IQR = interquartile range; LWV = Life Whisperer Viability; SCH = SwimCount Harvester; SU = Swim-up.

a Clinical and morphokinetic outcomes are presented according to the sperm DFI and the sperm selection method. Fertilization, usable blastocyst, embryo quality, and euploidy rates were calculated per mature oocyte or embryo biopsied. Morphokinetic parameters (t2–tB) represent the timing (in hours) of key embryonic developmental milestones recorded by time-lapse imaging. The LWV score indicates the embryo’s predicted probability of achieving pregnancy, as determined by an artificial intelligence–based evaluation. Comparisons between the SCH and SU groups were performed using the χ² or Mann–Whitney *U* tests as appropriate. *P* < .05 was considered statistically significant.

## Discussion

Numerous scientific articles have demonstrated that sperm quality plays a fundamental role in achieving successful clinical outcomes (34), with the total progressive motile sperm count and DNA fragmentation being one of the most reliable indicators for predicting male factor infertility (35, 36). When comparing sperm quality in samples processed using both sperm selection techniques, a decrease in the percentage of progressive motile spermatozoa was observed in the SCH group. However, this group also showed a statistically significant improvement in parameters such as concentration, total progressive motile sperm count, morphology, and chromatin structure stability. Furthermore, a significant decrease in DNA fragmentation was observed in the samples processed using SCH. This effect may be explained by the absence of centrifugation in this method because centrifugation has been consistently associated with increased generation of reactive oxygen species and, consequently, with higher levels of DNA fragmentation (37). Similar results have been reported in several studies (18, 20, 38). It is important to highlight that various studies used different microfluidic devices, leading to some variability in outcomes. Nevertheless, in general, sperm quality parameters consistently improve when using microfluidic devices (39, 40).

In the present study, although the fertilization rate was similar between the two groups, the usable blastocyst rate was higher in the SCH group. These results align with findings from other scientific publications employing different microfluidic-based sperm selection devices (13, 27, 41, 42). Embryo quality assessment on the basis of the ASEBIR criteria (33) showed a significantly higher number of good-quality blastocysts in the SCH group than in the Swim-up group. Several studies confirm that sperm selection using microfluidic devices enhances embryo quality from oocytes fertilized with sperm processed by this technique (13, 17, 28, 42, 43). However, the embryo quality assessment criteria employed by these investigators differed from those used in our study because they followed the classification proposed by Veeck and Zaninovic (44) in 2003. Nevertheless, these findings collectively indicate that microfluidics consistently improves embryo quality, even when evaluated according to different classification systems. Additionally, the euploidy rate per biopsied embryo was analyzed in patients undergoing ICSI treatment with preimplantation genetic testing for aneuploidy. In our study, no significant differences in the euploidy rate were found between the two groups, which is consistent with findings reported in previous publications (42, 45). However, other investigators have, indeed, reported a significant improvement in this parameter (22, 43, 46). Variations in reported outcomes may be explained by differences in study design. For instance, Kocur et al. (22) and Godiwala et al. (43) used nonsibling oocytes, potentially introducing variability in oocyte quality. Additionally, the study by Kocur et al. (22) included only samples with high sperm DNA fragmentation, whereas our study did not apply this exclusion criterion. Furthermore, those studies compared microfluidics with density gradient centrifugation, whereas our comparison was made against Swim-up.

It has been demonstrated that oocytes possess the ability to repair sperm DNA damage, particularly single-strand breaks (47, 48). In these scenarios, sperm DNA damage may be balanced by the oocyte’s repair capacity, thus enabling fertilization (49, 50, 51, 52). Nevertheless, this ability depends on the extent of genetic damage because highly fragmented DNA or low-quality oocytes can impair fertilization and subsequent embryo development (48, 53, 54, 55, 56). Consequently, we analyzed the effect of the microfluidic device according to two criteria: oocyte quality and sperm DNA fragmentation. The sample size was divided into two groups on the basis of the quality score of each oocyte. An AI score of ≥6 was adopted as the stratification threshold, coinciding with the cohort median and ensuring balanced and statistically comparable subgroups. Although Magenta defines the transition between low-medium and medium-high oocyte quality at a score of 5, the use of 6 as an operational cutoff was considered methodologically valid because it provided greater analytic power while remaining closely aligned with the clinically accepted threshold. Statistically significant differences were observed exclusively for embryo quality in the subgroup of lower-quality oocytes, with a higher number of good-quality embryos per mature oocyte in the SCH group. This finding could be explained by the interaction between oocyte quality and sperm quality, as previously described in the literature. Several studies have demonstrated that defects in sperm concentration, total progressive motility, and DNA fragmentation become less relevant with high-quality oocytes, yet these defects become clinically apparent when oocytes are aged or exhibit morphological abnormalities (49, 57, 58).

Additionally, we evaluated a subgroup stratified by the sperm DNA fragmentation index, comparing semen samples with less than 20% fragmentation before processing against those exceeding 20%. This threshold was selected on the basis of a systematic review and meta-analysis of 28 studies indicated that a threshold of 20% (in the terminal deoxynucleotidyl transferase dUTP nick end labeling protocol) best discriminates between fertile and infertile men, with an area under the curve of 0.844 (sensitivity, 79%; specificity, 86%) (47, 59). When analyzing the subgroup with sperm DNA fragmentation levels higher than 20% in fresh samples, microfluidic selection significantly improved the number of usable blastocysts and embryo quality, even though fertilization, euploidy, morphokinetics, and AI-based pregnancy scores were similar between groups. These results align with previous studies, showing microfluidic benefits in high-fragmentation cases, microfluidic platforms, by reducing sample handling and mechanical stress, better isolate sperm with intact DNA (42). In contrast, no significant differences were observed in the subgroup with sperm DNA fragmentation levels lower than 20%, likely because Swim-up techniques are already effective in such scenarios. Previous studies have demonstrated that conventional sperm selection techniques, such as Swim-up and density gradient centrifugation, fail to consistently improve DNA integrity in samples with high levels of DNA fragmentation (6, 42). These findings highlight the need for more advanced selection methods.

This study has several limitations. Primarily, because of low sperm concentrations in many samples, there was insufficient sperm concentration remaining after processing to assess sperm DNA fragmentation reliably. Additionally, subgroup analyses were limited by a small sample size, reducing the statistical power needed to detect significant differences. Another important limitation of this study is the absence of clinical data on implantation, pregnancy, and live birth. This is due to the short time interval between the completion of the study and the preparation of the manuscript, together with the inclusion of patients who underwent multiple embryo accumulation cycles, particularly in the context of preimplantation genetic testing for aneuploidy, which substantially limited the sample size available for the analysis of clinical outcomes. Nevertheless, this study also possesses notable strengths. Despite the use of sibling oocytes, an AI tool was employed to objectively assess the quality of each oocyte. Analysis of the scores generated by this tool showed that oocyte quality was statistically comparable between the SCH and Swim-up groups, both in the overall sample and across all evaluated subgroups. This homogeneity supports the robustness and reliability of the findings presented. Moreover, this is the first study evaluating the laboratory KPIs of the newly developed SCH device. Furthermore, morphokinetic parameters and pregnancy-prediction scores from the LWV AI tool were compared between the Swim-up sperm selection technique and microfluidics, a technique increasingly used in in vitro fertilization laboratories. Despite the growing adoption of microfluidics, morphokinetic parameters and AI-based predictions have rarely been studied in the context of these sperm selection methodologies.

## Conclusion

The SCH device improves sperm quality, except for motility and vitality. Moreover, the membrane-based microfluidic device increases the usable blastocyst rate and embryo quality while reducing the time to reach a blastocyst stage. However, no significant differences were observed in the fertilization rate, embryo euploidy, or LWV AI score.

Stratification of the data set revealed that embryo quality improved in the SCH group in patients with oocytes scoring below 6 on the AI assessment tool or with sperm DNA fragmentation above 20%. In the subgroup with increased DNA fragmentation, the SCH group also showed a higher rate of usable blastocysts.

## Declaration of Interests

F.M. reports honoraria and travel support from MotilityCount ApS and ASEBIR (Spanish Association for the Study of Reproductive Biology) Member of the Andrology Interest Group. L.C.-S. has nothing to disclose. A.d.A. has nothing to disclose. R.R.-E. has nothing to disclose. C.V. has nothing to disclose. J.A.R. has nothing to disclose. M.M. reports funding from Theramex (birth Grant) for the submitted work and honoraria from Merck, Vitrolife, MSD, Ferring, AIVF, Theramex, Gedeon Richter, Genea Biomedx, Fairtility, and Life Whisperer.
