# Supplementary material for: Novel sperm selection device on the basis of microfluidics improves usable blastocyst rates, embryo morphology, and morphokinetic patterns: sibling cohort prospective study
Source: F S Rep. 2025 Oct 6;6(4):436–45. doi: 10.1016/j.xfre.2025.09.008 (PMC12746890; doi:10.1016/j.xfre.2025.09.008)
Supplement: Supplementary file 1 [file mmc1.zip › xfre_535_Supplementary Figure and Tables_mmc1.odt]

Supplementary Table 1 Classification of embryo quality based on the morphology of the inner cell mass (ICM) and the trophectoderm (TE), 

according to the criteria established by ASEBIR (Spanish Association for the Study of Reproductive Biology).

A screenshot of a computer

AI-generated content may be incorrect.

A chart with different colors and letters

AI-generated content may be incorrect.A chart with different colored squares

AI-generated content may be incorrect.

Supplementary Table 2 Number of patients, mature oocytes, and blastocysts obtained according to the origin of the oocytes and the type of cycle used: without preimplantation genetic testing (ICSI) or with preimplantation genetic testing for aneuploidies (PGT-A). In addition, male age, fresh semen parameters, and semen abnormalities are detailed.

|  | Method | Patients | Oocytes | Blastocyst |
| --- | --- | --- | --- | --- |
| Total | SCH | 100 | 523 | 283 |
| Swim-up | 100 | 513 | 258 |
| Total | 100 | 1036 | 541 |
| Own Oocytes | SCH | 85 | 423 | 229 |
| Swim-up | 85 | 415 | 212 |
| Total | 85 | 838 | 441 |
| Donor Oocytes | SCH | 15 | 100 | 54 |
| Swim-up | 15 | 98 | 46 |
| Total | 15 | 198 | 100 |
| ICSI | SCH | 40 | 236 | 130 |
| Swim-up | 40 | 224 | 111 |
| Total | 40 | 460 | 241 |
| PGT-A | SCH | 60 | 287 | 153 |
| Swim-up | 60 | 289 | 147 |
| Total | 60 | 576 | 300 |
| Characteristics | Mean ± Sd | IQR |
| Male age | 40.00 ± 4.86 | 36.25 – 43.00 |
| Fresh semen parameters | Median | IQR |
| Volume (mL) | 2.00 | 1.20 - 3.00 |
| Concentration (x106/mL) | 32.00 | 15.00 - 54.25 |
| Progressive Motility (%) | 35.00 | 23.00 - 44.00 |
| Total Progressive Motile Sperm Count (x106) | 24.96 | 4.41 - 53.55 |
| Vitality (%) | 83.00 | 71.00 - 88.00 |
| Morphology (%) | 3.00 | 2.00 - 4.00 |
| Stable Chromatin Structure (%) | 82.00 | 76.00 - 85.00 |
| DNA Fragmentation (%) | 9.21 | 5.36 - 13.47 |
| Semen abnormalities | % (n/N) |
| Oligozoospermia | 23% (23/100) |
| Asthenozoospermia | 37% (37/100) |
| Teratozoospermia | 64,94% (50/77) |
| Oligoasthenoteratozoospermia | 7% (7/100) |
| Necrozoospermia | 10,45% (7/67) |
| Hipospermia | 25% (25/100) |
| High DNA Fragmentation (≥20%) | 18.18% (16/88) |
| At least one abnormal semen parameter | 85% (85/100) |

Supplementary Table 3 Comparison of scores from the Future Fertility Magenta artificial intelligence tool between the SwimCount™ Harvester (SCH) group and the swim-up group, across the different populations studied (oocyte origin and DNA fragmentation index).

|  | Subgroup | n (SCH) | SCH | n (SU) | Swim-up | p-Value |
| --- | --- | --- | --- | --- | --- | --- |
| Future Fertility Magenta Median [IQR] | Total | 489 | 6.00 [3.70–8.20] | 481 | 6.20 [3.70–8.10] | 0.6792 |
| Own oocytes | 389 | 6.20 [3.70–8.20] | 383 | 6.10 [3.80–8.10] | 0.7576 |
| Oocytes donated | 100 | 5.65 [3.80–8.10] | 98 | 6.35 [3.50–7.85] | 0.8012 |
| <20% DNA Fragmentation | 183 | 6.90 [4.50-8.40] | 166 | 6.65 [4.65-8.23] | 0.727 |
| ≥20% DNA Fragmentation | 44 | 7.00 [5.33-8.68] | 32 | 5.85 [4.75-8.85] | 0.453 |

Supplementary Table 4 Comparison of in vitro fertilization laboratory Key Performance Indicators (KPIs) between the SwimCount™ Harvester (SCH) and swim-up groups according to oocyte quality obtained using the Future Fertility Magenta artificial intelligence tool.

|  | Subgroup | SCH | Swim-up | χ² | p-Value |
| --- | --- | --- | --- | --- | --- |
| Fertilization rate per mature oocyte %(n/N) | Oocyte AI Score ≤6 | 68.98 % (169/245) | 66.67 % (156/234) | 0.294 | 0.588 |
| Oocyte AI Score ≤6 | 87.30 % (213/244) | 85.02 % (210/247) | 0.532 | 0.466 |
| Usable blastocyst rate per mature oocyte %(n/N) | Oocyte AI Score ≤6 | 34.29 % (84/245) | 26.50 % (62/234) | 3.428 | 0.064 |
| Oocyte AI Score ≤6 | 46.72 % (114/244) | 39.68 % (98/247) | 2.483 | 0.115 |
| Embryo quality rate per mature oocyte %(n/N) | Oocyte AI Score ≤6 | 26.53 % (65/245) | 17.52 % (41/234) | 5.637 | 0.018* |
| Oocyte AI Score ≤6 | 34.43 % (84/244) | 27.94 % (69/247) | 2.411 | 0.120 |
| Euploidy rate per embryo biopsied %(n/N) | Oocyte AI Score ≤6 | 11.54 % (15/130) | 10.32 % (13/126) | 0.098 | 0.754 |
| Oocyte AI Score ≤6 | 18.25 % (23/126) | 15.15 % (20/132) | 0.447 | 0.504 |
|  | Subgroup | n (SCH) | SCH | n (SU) | Swim-up | p-Value |
| t2 Median (hours) [IQR] | Oocyte AI Score ≤6 | 105 | 26,07 [24.03-28.37] | 91 | 25.62 [23.57-27.40] | 0.177 |
| Oocyte AI Score ≤6 | 149 | 25.25 [23.20-27,20] | 129 | 25.48 [23.91-27.15] | 0.252 |
| t3 Median (hours) [IQR] | Oocyte AI Score ≤6 | 105 | 36.50 [32.93-39.50] | 91 | 35.68 [32.67-38.01] | 0.431 |
| Oocyte AI Score ≤6 | 149 | 35.86 [32.10-38.57] | 129 | 36.46 [33.85-38.50] | 0.261 |
| t4 Median (hours) [IQR] | Oocyte AI Score ≤6 | 105 | 37.83 [35.17-40.95] | 91 | 37.20 [34.53-39.60] | 0.491 |
| Oocyte AI Score ≤6 | 149 | 37.25 [34.57-40.05] | 129 | 37.75 [35.16-39.52] | 0.335 |
| t5 Median (hours) [IQR] | Oocyte AI Score ≤6 | 105 | 47.93 [43.41-53.26] | 91 | 49.11 [44.00-51.97] | 0.246 |
| Oocyte AI Score ≤6 | 149 | 48.10 [43.84-52.38] | 129 | 49.30 [44.78-52.40] | 0.741 |
| tB Median (hours) [IQR] | Oocyte AI Score ≤6 | 105 | 105.63 [100.33-113.63] | 91 | 110.09 [102.60-115.72] | 0.077 |
| Oocyte AI Score ≤6 | 149 | 108.10 [102.05-115.30] | 129 | 109.03 [103.76-115.33] | 0.347 |
| Embryo AI Score (LWV) Median [IQR] | Oocyte AI Score ≤6 | 105 | 6.20 [2.65-8.10] | 91 | 5.00 [2.10-7.50] | 0.069 |
| Oocyte AI Score ≤6 | 149 | 5.10 [1.35-7.85] | 129 | 4.70 [1.60-7.40] | 0.774 |

Supplementary Figure 1 Representative image and schematic design of the SwimCount™ Harvester device, illustrating the two chambers separated by a membrane.

A close-up of a hand holding a plastic device

AI-generated content may be incorrect.
